# Effects of aquatic exercise on the improvement of lower-extremity motor function and quality of life in patients with Parkinson’s disease: A meta-analysis

**DOI:** 10.3389/fphys.2023.1066718

**Published:** 2023-02-03

**Authors:** Shengyu Dai, Haoteng Yuan, Jiahui Wang, Yuhang Yang, Shilin Wen

**Affiliations:** ^1^ Capital University of Physical Education and Sports, Beijing, China; ^2^ Shenzhen University, Shenzhen, Guangdong, China

**Keywords:** aquatic exercise, Parkinson’s disease, lower-extremity motor function, quality of life, meta analysis

## Abstract

**Objective:** To systematically evaluate the effect of aquatic exercise interventions on the improvement of lower-extremity motor function and quality of life in patients with Parkinson’s disease.

**Methods:** Two researchers independently searched the literature using the PubMed, Web of Science, Embase, and Cochrane Library databases. The search period was from the establishment of the database to December 2021. The subject heading search included “hydrotherapy,” “hydro therapies,” “hydro therapeutics,” “water therapy,” “aquatic exercise therapy,” “aquatic therapy,” “water-based exercise,” “Parkinson,” “Parkinson disease,” “Parkinson’s disease,” “Parkinson’s syndrome,” “primary Parkinsonism,” “paralysis agitans,” and “randomized controlled trial (RCT).”

**Result:** A total of 698 articles were retrieved from the four databases by searching for subject headings, and 10 RCT articles were finally included. The balance ability of aquatic exercise in patients with Parkinson’s disease (weighted mean differences [WMD] = 2.234, 95% CI: 1.112–3.357, Z = 3.9, *p* < 0.01), walking ability (WMD = −0.911, 95% CI: −1.581 to −0.241, Z = 2.67, *p* < 0.01), and quality of life (WMD = −5.057, 95% CI: −9.610 to −0.504, Z = 2.18, *p* = 0.029) were improved, but there was no significant difference in motor function (WMD = −0.328, 95% CI: −1.781 to 1.125, Z = 0.44, *p* = 0.658).

**Conclusion:** Compared with conventional rehabilitation therapy, aquatic exercise can effectively improve balance, walking ability, and quality of life in patients with Parkinson’s disease. However, it had no obvious effect on improving motor function. This study was limited by the number and quality of the included studies, and more high-quality studies are needed to verify this.

**Systematic Review Registration:** https://www.crd.york.ac.uk/prospero/, identifier CRD42022365103.

## 1 Introduction

Parkinson’s disease (PD) was first described as a clinical syndrome by James Parkinson in 1817 (Jankovic and Tan, 2020), and is a disorder characterized by involuntary behavior. Currently, PD is the second most common neurodegenerative disease in the world, with an increasing incidence annually. PD is mainly characterized by resting tremors, bradykinesia, stiffness, postural instability, and various other non-motor symptoms.

The prevalence of PD is increasing faster than that of other neurological diseases (Mele et al., 2020). The global prevalence of PD is 0.3%, accounting for 1%–2% of people over 65 years of age (Wirdefeldt et al., 2011). According to research statistics, the prevalence of PD worldwide is expected to double to more than 14 million people by 2040 (Dorsey and Bloem, 2018). PD, a degenerative disease of the central nervous system with a low mortality and high disability rate, brings great inconvenience to patients’ lives, seriously threatens the physical and mental health of the elderly population, and places a burden on society. This has become a public health problem that needs to be solved urgently (Rana et al., 2022).

Currently, medication remains the main treatment for patients with PD. Many studies have confirmed that exercise therapy is also considered to be one of the effective rehabilitation methods to treat patients with PD. The rehabilitation effect of exercise therapy on the motor function of patients with PD has been proven to improve balance (King et al., 2015) and reduce falls (Shen et al., 2016; Mak et al., 2017). Extensive research has shown that exercise interventions can significantly improve the symptoms of motor disorders and the quality of life of patients with PD through potential mechanisms such as neuroprotective and neuroplastic effects.

Aquatic exercise is widely used in the rehabilitation of neurological diseases (Castro-Sánchez et al., 2012; Marinho-Buzelli et al., 2019; Saleh et al., 2019), and refers to a method of exercise or rehabilitation training and treatment in water, which can relieve the symptoms of patients and improve motor function (Higgins et al., 2011). Aquatic exercise to improve the challenges of PD has been a hot topic of interest for researchers in recent years, but the effects of aquatic exercise on PD patients are still controversial.

Previous studies have shown that aquatic therapy (Giladi, 2009; Ayán Pérez and Cancela, 2014), can improve motor activity, gait, quality of life (Carroll et al., 2017; de la Cruz, 2017; Palamara et al., 2017), and postural stability in patients with mild to moderate PD (Suomi and Koceja, 2000; Devereux et al., 2005; Arnold et al., 2008; Katsura et al., 2010; Elbar et al., 2013). Chae et al. (2020); Li et al. (2017) showed that rehabilitation interventions using the aquatic environment can significantly improve balance function and lower-limb muscle strength in patients, while other studies have also confirmed that aquatic exercise training leads to significant improvements in the health status and activities of daily living in patients with PD (Pellecchia et al., 2004; Perez-Lloret et al., 2014; Volpe et al., 2014). However, Volpe et al. (Volpe et al., 2020) investigated the extent to which underwater gait training improved muscle activity in patients with PD. The study included 10 patients with PD and 10 healthy people, and the walking stride and speed of the PD patients improved on day four after the intervention. Contrastingly, Pinto et al. (Pinto et al., 2019) found that underwater exercise did not significantly improve the quality of life of PD patients compared to traditional land-based exercise training in a systematic review of non-randomized controlled trials. We aimed to conduct a meta-analysis of the literature related to aquatic exercise to improve lower-extremity motor function and quality of life in patients with PD to further explore the application of aquatic interventions in the treatment thereof.

## 2 Materials and methods

### 2.1 Literature search

Two researchers independently searched the PubMed, Web of Science, Embase, and Cochrane Library databases. The retrieval period was from the establishment of the database to December, 2021. The search terms included “hydrotherapy,” “hydrotherapies,” “hydrotherapeutics,” “water training,” “aquatic exercise,” “water exercise therapy,” “water therapy,” “aquatic exercise therapy,” “aquatic therapy,” “water-based exercise,” “Parkinson’s disease,” “Parkinson’s syndrome,” “paralysis tremor,” “Parkinson’s syndrome,” “primary Parkinsonism,” “paralysis agitans,” and “randomized controlled trial (RCT).”

### 2.2 Inclusion and exclusion criteria

The following criteria were required for the included studies: 1) the literature was in Chinese and English full-text; 2) the experimental group involved aquatic exercise; 3) the study was a RCT; 4) the experimental subjects were PD patients (Hoehn–Yahr clinical stage I–IV, PD duration >1 year) without other diseases or complications; 5) the patients were aged >18 years; and 6) the outcomes included the Berg Balance Scale (BBS), timed up-and-go test (TUGT), Parkinson’s Disease Unified Rating Scale III (UPDRS III), and the Parkinson’s disease questionnaire (PDQ)-39.

We excluded studies with the following criteria: 1) the subjects were non-Parkinsonian; 2) the study was a meta-analysis; 3) the experimental design was a non-RCT; 4) the intervention was of non-water exercise; 5) repeatedly published or irrelevant literature; and 6) literature for which original data could not be obtained.

### 2.3 Literature screening and data extraction

Literature screening and data extraction were independently performed by two investigators according to the inclusion and exclusion criteria. For any disagreements, both parties discussed or consulted a third researcher to assist in adjudication. The following data were extracted: title; first author; publication year; sample size; intervention; intervention time; frequency; and outcome indicators.

### 2.4 Risk assessment of bias in the included literature

The quality of the included literature was evaluated according to the literature quality assessment standard manual recommended by the Cochrane Manual 5.1.0 (Higgins et al., 2019). The manual evaluates seven types of bias: random sequence generation (selection bias); allocation concealment (selection bias); blinding of participants and personnel (performance bias); blinding of outcome assessment (detection bias); incomplete outcome data (attrition bias); selective reporting (reporting bias); and any other bias. The authors judged the risk of bias of the included literature as low, unclear, or high risk. If there was a dispute, a third party was involved in the discussion until a consensus was reached.

### 2.5 Statistical analysis

A meta-analysis of the data was performed using the Stata 15 software. Effect sizes were analyzed using weighted mean differences (WMD) or standardized mean differences. An effect size of *p* ≤ 0.05 indicated statistical significance. The 95% confidence intervals for the fixed- and random-effects models were calculated. When I^2^ < 50%, the fixed-effects model was used; otherwise, the random-effects model was used for analysis. The articles were analyzed for publication bias using Egger’s test to ensure the reliability of the data.

## 3 Results

### 3.1 Literature screening

**FIGURE 1:**
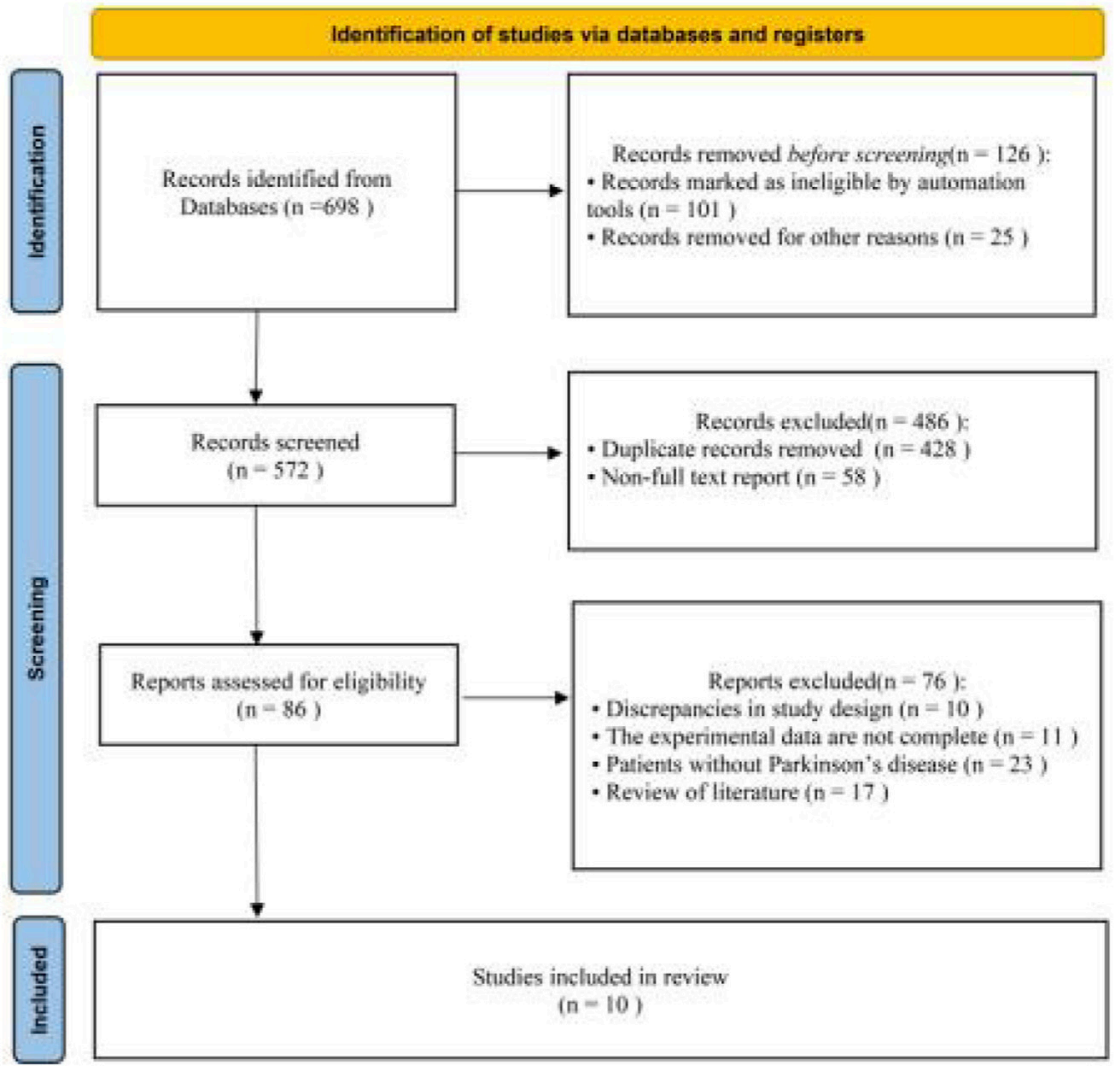
Flowchart of the literature screening process.

A total of 698 studies were obtained through a preliminary search. After removing duplicates and excluding studies based on our inclusion and exclusion criteria, 10 RCTs were included in the meta-analysis. The literature screening process is illustrated in Figure 1.

### 3.2 Basic information of the included literature

Ten articles (Carroll et al., 2017; de la Cruz, 2017; Palamara et al., 2017; Volpe et al., 2014; Vivas et al., 2011; Volpe et al., 2017a; Volpe et al., 2017b; Kurt et al., 2018; Clerici et al., 2019; da Silva and Israel, 2019) were included in this study. A total of 298 patients with PD were included, with 152 in the experimental group and 146 in the control group. Hoehn–Yahr stage (H&Y) was used to evaluate the degree of PD. The outcome indicators were the BBS, TUGT, UPDRS III, and PDQ-39. Basic information on the included studies is presented in Table 1.

**TABLE 1:** Basic characteristics of the included literature.

First author	Year	Country	Age (T/C)	Cases (T/C)	Intervention cycle (week)	Intervention frequency (times/week)	Intervention time (min/times)	Specific measures	Control group content	Outcome measures
Vivas et al. (2011)	2011	Spain	65.67 ± 3.67/68.33 ± 6.92	5/6	4	2	45	Environmental requirements: water depth 1.3 m, water temperature 32 °C; aquatic exercise: warm up, 10 min (water walking and floating training), water training for 35 min (trunk movement for 15 min, postural stability for 10 min, self-transfer exercise for 10 min)	Land drill	1, 2, 3
Volpe et al. (2014)	2014	Italy	68.00 ± 7.00/66.00 ± 8.00	17/17	8	5	60	Aquatic exercise: warm up for 10 min, water balance training for 40 min, relaxation for 10 min	Land drill	1, 2, 3, 4
Carroll et al. (2017)	2017	Ireland	69.50 ± 1.75/74.00 ± 6.01	10/8	6	2	45	Environmental requirements: water depth 0.6–1.3 m, water temperature 32 °C; aquatic exercise + routine treatment: warm up for 10 min (aerobic exercise, stretching exercise), gait training for 20 min, strength training for 10 min, relaxation activity for 5 min	Routine treatment	3, 4
Palamara et al. (2017)	2017	Italy	70.90 ± 5.70/70.80 ± 5.30	17/17	4	3	60	Environmental requirements: water temperature 33–34 °C; MIRT + aquatic exercise: warm up for 10 min, water training for 30–45 min (trunk movement training, dynamic and static exercises, balance training), relaxation activity for 5–10 min	MIRT + land drill	1, 2, 3, 4
de la Cruz (2017)	2018	Spain	66.80 ± 5.27/67.53 ± 9.89	15/15	11	2	45	Environmental requirements: water depth 1.1 m, water temperature 30 °C; water Tai chi training: warm up, tai chi training for 35 min (torso rotation, standing balance, one leg balance), relaxation activities	Land drill	1, 2, 3
Volpe et al. (2017a)	2017	Italy	70.60 ± 7.80/70.00 ± 7.80	15/15	8	5	60	Aquatic exercise: warm up exercises, postural exercises (perturbation-based balance training), relaxation activities	Land drill	1, 2, 3, 4
Volpe et al. (2017b)	2017	Italy	76.7 ± 4.0/78.4 ± 4.6	12/12	3	7	60	Aquatic exercise: warm up for 10 min, walking for 40 min, relaxation for 10 min	Land drill	1, 2, 3, 4
Kurt et al. (2018)	2018	Turkey	62.41 ± 6.76/63.61 ± 7.18	20/20	5	5	60	Environmental requirements: water depth 1.2 m, water temperature 32 °C; aquatic exercise: warm up, Tai chi training, relaxation activities	Land drill	1, 2, 3, 4
Clerici et al. (2019)	2019	Italy	67.00 ± 8.00/67.00 ± 11.00	27/25	4	3	60	Environmental requirements: water temperature 33–34 °C; MIRT + aquatic exercise: warm up for 10 min, water training for 30 min (ankle and hip joint training, dynamic and static exercises, balance training), relaxation for 10 min	MIRT + land drill	1, 2, 3
da Silva and Israel (2019)	2019	Brazil	63.12 ± 13.61/64.23 ± 13.45	14/11	10	2	60	Aquatic exercise: measurement of physical signs for 10 min, water training for 50 min (walking exercise, rotation and dual task activities)	Blank control	1, 2

The outcome indicators were the following: 1) the BBS; 2) TUGT; 3) UPDRS III; and 4) PDQ-39.

### 3.3 Quality assessment

**FIGURE 2:**
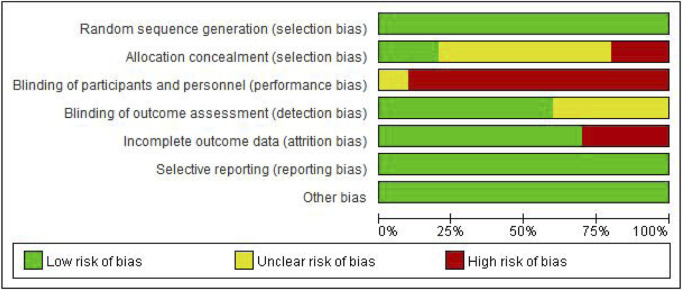
Schematic diagram of risk bias.

According to the Cochrane Collaboration risk-of-bias assessment tool standard, the 10 RCTs included were of moderate quality (Figure 2).

### 3.4 Results of meta-analysis

#### 3.4.1 BBS

**FIGURE 3:**
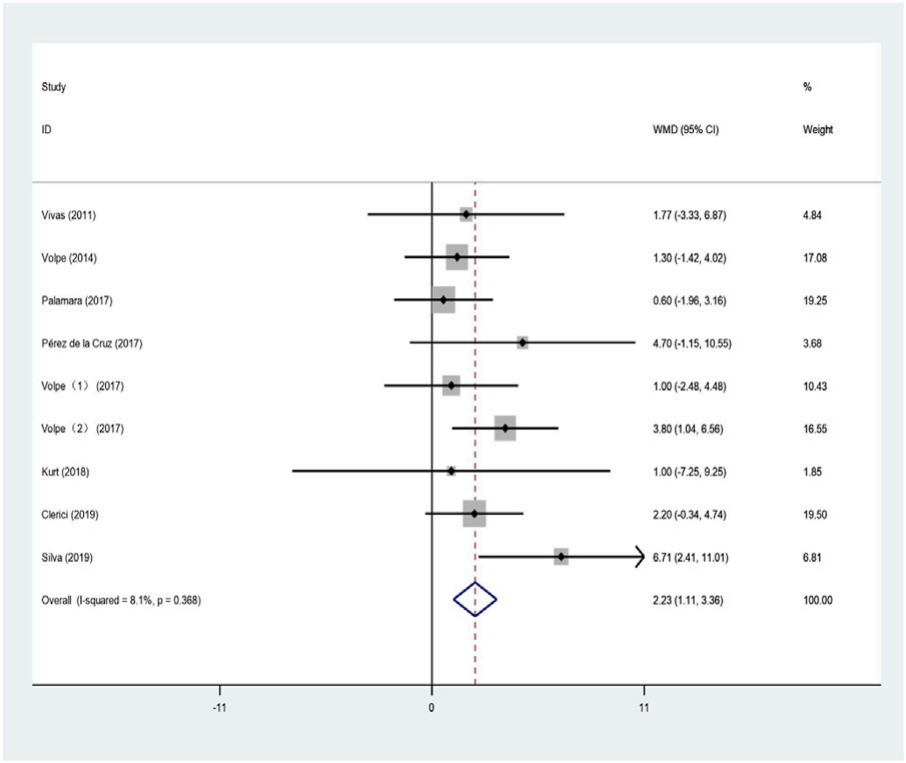
Effects of aquatic exercise on balance function in PD patients.

The BBS is a comprehensive scale composed of 14 indicators used to evaluate balance and fall risk in the elderly (Berg et al., 1989). The higher the score, the lower the risk of fall. Nine studies used the BBS to evaluate the effect of aquatic exercise on balance function in patients with PD. The results of combining data from the included studies for the meta-analysis are shown in Figure 3.

The heterogeneity test indicated large heterogeneity (I^2^ = 8.1%, *p* = 0.368); therefore, the fixed-effects model was used for analysis. This indicates that, compared with land-based training or routine nursing, aquatic exercise had a positive effect on balance function in patients (WMD = 2.234; 95% CI: 1.112–3.357; Z = 3.9; *p* < 0.01).

#### 3.4.2 TUGT

This test is often used to assess balance and physical fitness. The subject sits in a chair with armrests, stands up from the chair, walks forward to a straight line 3 m after hearing a command, turns around, walks back to the chair, turns around, and sits down smoothly. The tester records the time to complete the entire process. The shorter the time, the better the balance and physical ability (Benecke et al., 1987).

**FIGURE 4:**
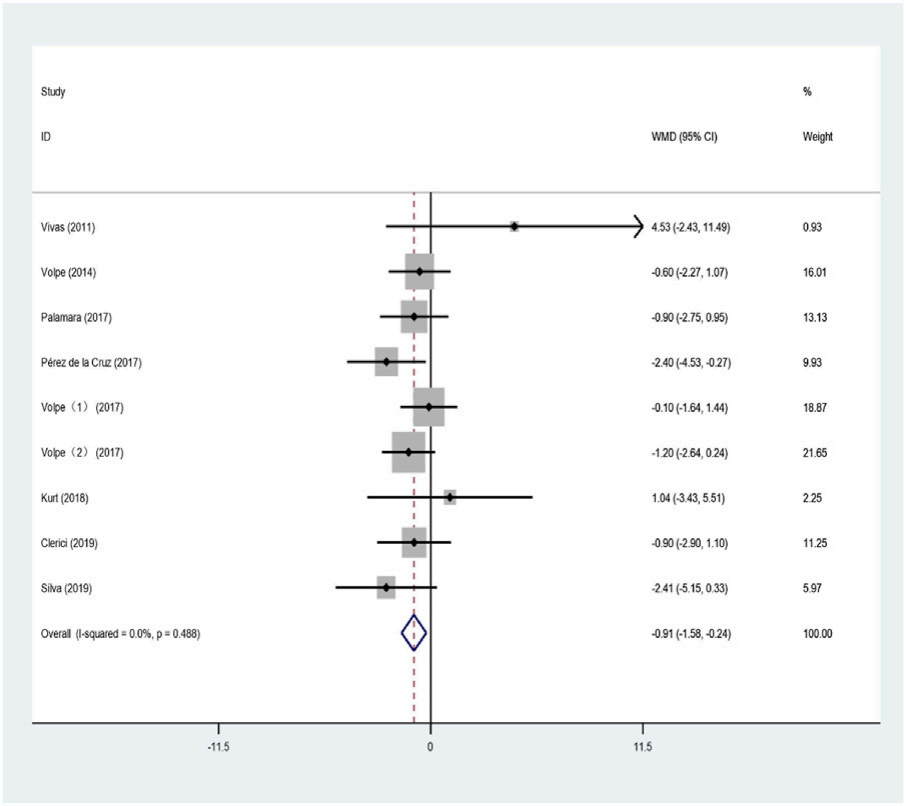
Effects of aquatic exercise on walking ability of PD patients.

Nine studies used TUGT to evaluate the effect of aquatic exercise on the walking ability of patients with PD. The results were analyzed by combining the data from the included studies in a meta-analysis, as shown in Figure 4. The heterogeneity test indicated small heterogeneity (I^2^ = 0%, *p* = 0.488); therefore, the fixed-effects model was used for analysis. This indicated that aquatic exercise can significantly improve the walking ability of patients with PD compared with land-based training or routine nursing (WMD = −0.911; 95% CI: −1.581 to −0.241; Z = 2.67; *p* < 0.01).

#### 2.4.3 UPDRS III

**FIGURE 5:**
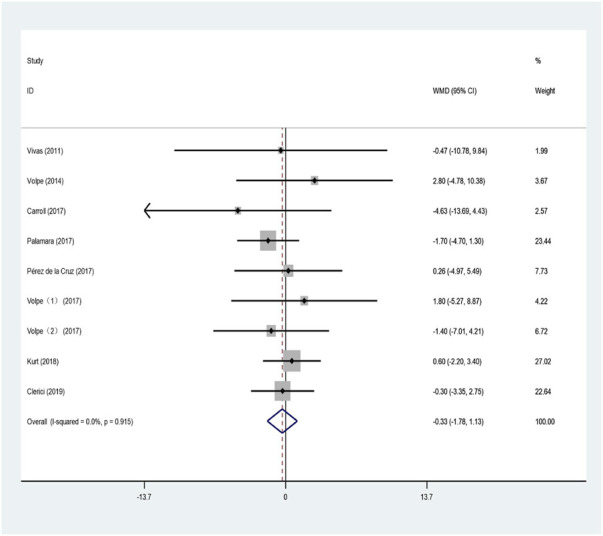
Influence of aquatic exercise on motor function of PD patients.

The UPDRS III is a commonly used scale to evaluate the quality of life of patients with PD, and is used to evaluate the motor function and signs of patients (Vivas et al., 2011). Ten studies used the UPDRS Ⅲ score to evaluate the effect of aquatic exercise on lower-extremity muscle strength in patients with PD. The meta-analysis was performed by combining data from the included studies, and the results are shown in Figure 5.

The heterogeneity test showed that the heterogeneity was small (I^2^ = 0%, *p* = 0.915); therefore, the fixed-effects model was used for the analysis. This indicated that, compared with land-based training or routine nursing groups, aquatic exercise has no significant improvement effect on the motor function of patients with PD (WMD = −0.328; 95% CI: −1.781 to 1.125; Z = 0.44; *p* < 0.01).

#### 2.4.4 Quality of life

The PDQ-39 is composed of eight items and 39 questions. A lower score indicates better quality of life is (Villegas and Israel, 2014). Five studies used the PDQ-39 to evaluate the effects of aquatic exercise on the quality of life of patients with Parkinson’s.

**FIGURE 6:**
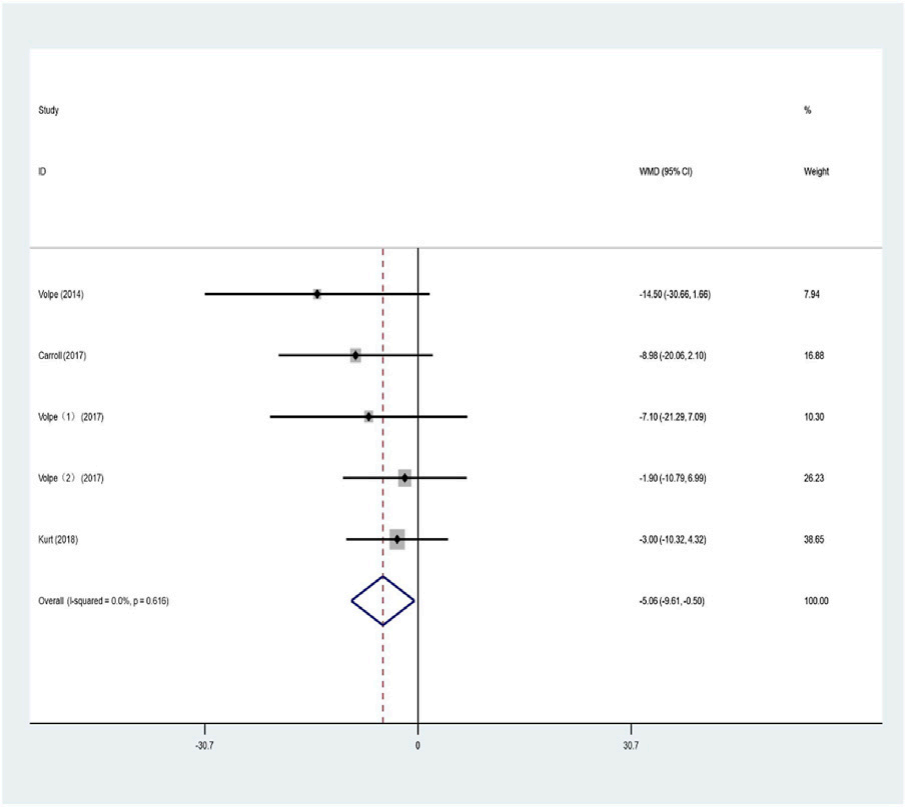
Effects of aquatic exercise on quality of life of PD patients.

The results were analyzed by combining the data from the included studies in a meta-analysis, as shown in Figure 6. The heterogeneity test revealed no heterogeneity (I^2^ = 0%, *p* = 0.616); therefore, the fixed-effects model was used for the analysis. This indicated that, compared with land-based training or routine nursing groups, aquatic exercise has a significant improvement effect on the quality of life of patients with PD (WMD = −5.057; 95% CI: −9.610 to −0.504; Z = 2.18; *p* = 0.029).

## 4 Discussion

A total of 698 articles were retrieved from the four databases by searching for the subject headings, and 10 RCT articles were finally included. The balance ability of aquatic exercise in patients with PD, walking ability, and quality of life improved, but there was no significant difference in motor function.

In recent years, the incidence of PD and its related complications has increased worldwide. PD not only involves motor and behavioral disorders but also has certain effects on cognitive function and mood. The main characteristics of motor behavioral disorders are reduced movement, stiffness, tremors, postural instability, and abnormal gait. Numerous studies have confirmed that exercise training can improve symptoms, delay the progression of PD, and promote motor function rehabilitation (Allen et al., 2011; Combs et al., 2011; Duncan and Earhart, 2012; Tseng et al., 2015; Zhang et al., 2015).

In the process of exploring exercise interventions to improve the symptoms of PD, aquatic exercise has been regarded as an important treatment strategy and has demonstrated some unique advantages. Aquatic exercise therapy is a treatment that makes use of the buoyancy, resistance, hydrostatic pressure, and fluidity of water to allow PD patients to perform exercise to train muscle strength, control, and coordination to relieve their symptoms or improve motor function (Towler et al., 1987; Pinto et al., 2019). We adopted the measurement of Parkinson’s patients with lower-extremity motor function and quality of life indexes; the commonly used evaluations after the relevant indicators, according to the results of the aquatic exercise, can effectively improve balance and walking ability, and improve patient quality of life. However, the control group showed no significant improvement in sports ability. Pinto (Pinto et al., 2019) and Cugusi (Cugusi et al., 2019) conducted systematic evaluations and meta-analyses that were also consistent with the results of this study, which verified the effectiveness of aquatic exercise therapy in the improvement of PD symptoms.

The consequences of impaired balance function in patients with PD are manifested in defects in control support ability during daily activities (Johnell et al., 1992; Sato et al., 2001), which is an important cause of falls in PD patients, and serious cases may lead to fractures and death (Foreman et al., 2011). Improving the balance of patients can reduce the risk of balance-related falls (Wong-Yu and Mak, 2019). The BBS is often used to assess balance and fall risk in older adults (Berg et al., 1989). In this study, the BBS was used to evaluate the balance function of PD patients. The results indicated that aquatic exercise had a positive effect on balance compared with land-based training or routine nursing (WMD = 2.234; 95% CI: 1.112–3.357; Z = 3.9; *p* < 0.01).

The European physiotherapy guidelines for exercise in PD recommend that treatment should be maintained for 8 weeks, three times a week for 45 min. According to the studies included here, the length of the aquatic exercise intervention was found to be 45–60 min and the duration of the intervention 3–11 weeks (Domingos et al., 2018). Although some studies did not meet the criteria of the European physiotherapy guidelines for exercise in PD, all included studies showed some improvement in the symptoms of PD patients through the intervention. Zhu et al. (Zhu et al., 2018) compared 46 cases of Hoehn–Yahr stage 2–3 PD patients who were randomly assigned to hydrotherapy or disorder hydrotherapy groups. All subjects received 30 min of aquatic therapy, five times a week for 6 weeks. The results of the study showed that both aquatic therapy regimens improved balance in PD patients, and gait freeze was significantly relieved after this training, which indicates that obstacle aquatic therapy was more effective than traditional aquatic therapy. Therefore, aquatic exercise is a feasible treatment for balance dysfunction in PD patients. The results of the studies by Volpe et al. (2014); Kurt et al. (2018) were similar to ours. Saleh and Masiero’s study also showed that aquatic exercise enhances proprioception and promotes balance in patients with PD (Masiero et al., 2019; Saleh et al., 2019). Therefore, when performing aquatic exercise interventions in PD patients, researchers should use the appropriate period, frequency, and duration of interventions to achieve greater effectiveness and scientific validity of aquatic exercise interventions for PD patients.

TUGT is a walking ability test that involves standing up, walking, turning, and sitting down sequentially (Benecke et al., 1987). Studies use the TUGT to evaluate the walking function of PD patients, and a longer time indicated worse walking function. A meta-analysis of the included literature indicated that aquatic exercise significantly improved walking ability in patients with PD compared with land-based training or routine nursing groups (WMD = −0.911; 95% CI: −1.581 to −0.241; Z = 2.67; *p* < 0.01). Studies of patients with PD have found that three (Palamara et al., 2017) to five treatments per week (Volpe et al., 2014; Kurt et al., 2018; Zhu et al., 2018) result in greater improvements in functional activity and gait in the aquatic exercise group compared to land-based training. This suggests that, to obtain clinically meaningful improvements in exercise in PD, treatment should be performed at least three times per week. Athletes with PD show reduced stride length and gait speed after injury (Tan et al., 2011), and Carroll proposed that the degree of gait change is related to the length of the intervention time (Carroll et al., 2017). Studies have found that aquatic exercise improves postural control, gait parameters (speed, rhythm, stride length), and hip motion in patients with PD (Volpe et al., 2017b), especially hip rotation and knee and ankle flexion (Cancela et al., 2015).

The buoyancy of water can reduce the load on both lower extremities and the impact of the ground reaction force on the joints, which can effectively improve the stability of the foot and walking ability (Masumoto et al., 2013). Paula found that the displacement angles of the hip, knee, and ankle joints of Parkinson’s patients increased, which increased the stability of posture and reflected the increase in physical resistance in the hydrotherapy environment (Rodriguez et al., 2013). Several studies have shown that aquatic exercise therapy can significantly improve gait variability (walking speed, stride length, and single and double support times) in patients with PD, which is significant for rehabilitation gait training (Ayán et al., 2014; Carroll et al., 2017; Zhu et al., 2018; Clerici et al., 2019).

Extensive studies have shown that exercise interventions can effectively improve movement disorders and promote the rehabilitation of motor function in patients with Parkinson’s (Tseng et al., 2015; Zhang et al., 2015; Ni et al., 2016; Taghizadeh et al., 2018). Aquatic exercise has gradually been applied as a new type of rehabilitation therapy. Vivas tested 11 patients with stage 2 or stage 3 idiopathic PD who were trained for a period of 4 weeks of individual training for 45 min, twice a week. The results showed that the UPDRS III score of the aquatic exercise group significantly increased, and the motor activity of PD patients improved (Vivas et al., 2011). Aquatic exercise improves body control and movement as well as flexibility in patients with PD, being able to constantly adjust their behavior and motor control in the water, keeping the body in a relaxed and physically extended state with slow and continuous movements (Villegas and Israel, 2014; Pérez-de la Cruz, 2019). In this study, the UPDRS III score was used to evaluate the effect of aquatic exercise on lower-limb muscle strength in patients with Parkinson’s. The results of the meta-analysis indicated that, compared with land-based training or routine nursing, aquatic exercise had no obvious improvement effect on the motor activity of patients with PD (WMD = −0.328; 95% CI: −1.781 to 1.125; Z = 0.44; *p* = 0.658). This differs from the results of a study by Terrens et al. (2018), and may be related to the small number of included studies, age of the subjects, different years of disease, and long-term exercise.

The PDQ-39 scale can be used to evaluate the quality of life of patients with PD, with lower scores indicating better quality of life (Villegas and Israel, 2014). In this study, the PDQ-39 was used to evaluate the effects of aquatic exercise on the quality of life of patients with Parkinson’s. The meta-analysis indicated that, compared with land-based training or routine nursing groups, aquatic exercise had a significant improvement effect on the quality of life of patients (WMD = −5.057, 95% CI: −9.610 to −0.504; *p* = 0.029), which is the same as the study results of Ayán and Cancela. (2012); Cugusi et al. (2019). Numerous studies have also shown a positive effect of aquatic exercise in improving the quality of life of patients with PD (Ayán and Cancela, 2012; Shahmohammadi et al., 2017), with significant improvements in pain, depression, and quality of life indicators (Pérez-de la Cruz, 2019), which are more conducive to better rehabilitation effects (Shulman et al., 2002; Volpe et al., 2014; Volpe et al., 2017a; Kurt et al., 2018). However, the results of the meta-analysis by Pinto et al. (2019) are contrary to the results of this study, which may be related to the small number of included studies.

Aquatic exercise may improve the quality of life of PD patients due to the following reasons: 1) aquatic exercise is unique in that individuals can be subjected to the pressure of the water, relieving muscle fatigue and reducing pain during exercise (Morris, 2010; Vivas et al., 2011; Volpe et al., 2014); 2) aquatic exercise can improve the balance function of patients, expand the range of activities, and thus improve the ability of daily activities (Pellecchia et al., 2004; Volpe et al., 2017a); 3) regular physical exercise can enhance self-efficacy, reduce anxiety and depression, improve mood and self-esteem, relieve stress, and improve quality of life (Peluso and De Andrade, 2005; Pérez-de la Cruz, 2019).

Furthermore, aquatic exercise can improve the symptoms of Parkinson’s patients by the following possible mechanisms. 1) Water has specific physical properties and the density of water is higher than that of air; patients can undergo resistance from all directions, which can effectively strengthen and exercise the patient postural control ability, improve muscle strength, muscular endurance, and joint mobility, thus improving balance function. The use of water buoyancy can reduce the contraction load of the lower limbs and the impact force on the joints, and reduce the difficulty of movement and the risk of walking training. Previous studies have confirmed that aquatic exercises can more effectively control posture and maintain balance (Becker, 2009; Zhang et al., 2016; Zhu et al., 2018). 2) The warming effect of water can stimulate temperature receptors and activate cortical sensory-related areas, regulate oxidative stress, increase the reaction between nerves and hippocampus (Silva Germanos et al., 2019; Dani et al., 2020), increase the activity of cortical sensory and motor areas, and promote sensorimotor integration. It also increases the extensibility of collagen tissue (Ardıç et al., 2007), inhibits the over-excitability of the stretch reflex, improves the flexibility of muscle function, relieves stiffness, and increases the self-confidence and training enthusiasm of patients.

This study had some limitations. First, the number of included studies was small, with a lack of long-term follow-up and long-term traceability. Second, the intervention programs were not uniform, and the intervention duration, frequency, and temperature of the pool may have affected the results. Third, the included studies did not clearly indicate the implementation of allocation hiding and blinding, which may have an impact on the credibility of the results.

## 5 Conclusion

Aquatic exercise can effectively improve the balance function, walking ability, and quality of life of patients with PD, but the improvement effect on the motor function of patients is not significant, limited by the number and quality of the included studies. Further studies are required, with the inclusion of high-quality RCTs.

## Data Availability

The original contributions presented in the study are included in the article/supplementary material, further inquiries can be directed to the corresponding authors.
